# Regulation of overexpressed efflux pump encoding genes by cinnamon oil and trimethoprim to abolish carbapenem-resistant *Acinetobacter baumannii* clinical strains

**DOI:** 10.1186/s12866-024-03194-8

**Published:** 2024-02-08

**Authors:** Neveen M. Saleh, Hadeer Ezzat, Gharieb S. El-Sayyad, Hamdallah Zedan

**Affiliations:** 1 Department of Microbiology, Egyptian Drug Authority (former National Organization for Drug Control and Research (NODCAR), Giza, Egypt; 2 Microbiology and Immunology Department, Faculty of Pharmacy, Ahram Canadian University (ACU), 6th October City, Giza, Egypt; 3 Department of Microbiology and Immunology, Faculty of Pharmacy, Galala University, New Galala City, Suez, Egypt; 4 Drug Microbiology Lab., Drug Radiation Research Department, National Center for Radiation Research and Technology (NCRRT), Egyptian Atomic Energy Authority (EAEA), Cairo, Egypt; 5 Department of Microbiology and Immunology, Faculty of Pharmacy, Cairo University, Cairo, Egypt

**Keywords:** *Acinetobacter baumannii*, Efflux pump, AdeJKL, Efflux pump inhibitors, Cinnamomum oil

## Abstract

Resistance mechanisms are a shelter for *Acinetobacter baumannii* to adapt to our environment which causes difficulty for the infections to be treated and WHO declares this organism on the top of pathogens priority for new drug development. The most common mechanism that develops drug resistance is the overexpression of the efflux pump, especially Resistance-nodulation-cell division (RND) family, to almost most antibiotics. The study is designed to detect RND efflux pump genes in *A. baumannii*, and its correlation to multidrug resistance, in particular, the carbapenems resistance *Acinetobacter* *baumannii* (CRAB), and using different inhibitors that restore the antibiotic susceptibility of imipenem. Clinical *A. baumannii* isolates were recovered from different Egyptian hospitals in Intensive care unit (ICU). The expression of genes in two strains was analyzed using RT-PCR before and after inhibitor treatment. About 100 clinical *A. baumannii* isolates were recovered and identified and recorded as MDR strains with 75% strains resistant to imipenem. *adeB, adeC, adeK,* and *adeJ* were detected in thirty- seven the carbapenems resistance *Acinetobacter* *baumannii* (CRAB) strains. *Cinnamomum verum* oil, Trimethoprim, and Omeprazole was promising inhibitor against 90% of the carbapenems resistance *Acinetobacter* *baumannii* (CRAB) strains with a 2-6-fold decrease in imipenem MIC. Downregulation of four genes was associated with the addition of those inhibitors to imipenem for two the carbapenems resistance *Acinetobacter* *baumannii* (CRAB) (ACN15 and ACN99) strains, and the effect was confirmed in 24 h killing kinetics. Our investigation points to the carbapenems resistance *Acinetobacter* *baumannii* (CRAB) strain’s prevalence in Egyptian hospitals with the idea to revive the imipenem activity using natural and chemical drugs as inhibitors that possessed high synergistic activity.

**Supplementary Information:**

The online version contains supplementary material available at 10.1186/s12866-024-03194-8.

## Introduction

*Acinetobacter baumannii* is a leading cause of nosocomial infections that severely threaten public health. It is an opportunistic gram-negative coccobacillus that demonstrates exquisite survival under various environmental conditions and intrinsic resistance to routinely prescribed antibiotics [1]. It belongs to ESKAPE pathogens *(Enterococcus faecium, Staphylococcus aureus, Klebsiella pneumoniae, A. baumannii, Pseudomonas aeruginosa, and Enterobacter spp*.) that contributed to global health concern due to increasing antibiotic resistance especially carbapenem resistance *A. baumannii* (CRAB) that associated to co-resistance to other antibiotics [2, 3]. The drastic increment in the predominance of infectious diseases induced by multidrug-resistant *A. baumannii* has postures an immense concern since these pathogens create severe challenges for patients through their propensity for developing antibiotic resistance extremely rapidly. Furthermore, diseases caused by these strains are frequently related to raised mortality rates, drawn-out hospitalization, and costs, and; therefore, it has been designated as a “red alert” human pathogen [1, 4]. In addition, the carbapenem and polymyxin widespread resistance in Southeast Asia nations represents a continuing risk and increases the threat of infections resistant to both classes [5].

Mechanisms of Β-lactam resistance are varied and involve many fully identified genes. A well-known, identified mechanism involves decreased accumulation of antibiotics inside bacterial cells through the efflux pump and/or decreased permeability (outer membrane proteins). Efflux is known to be a ubiquitous mechanism associated with antibiotics resistance [6] outside the bacterial cell and nowadays, because of its non-specificity, efflux pumps are becoming relevant due to their broad substrate profile [7], leading to cross-resistance with multiple antibiotics, and can interact synergistically with other resistance mechanisms to increase the level of resistance [8, 9].

Unfortunately, many efflux pumps have been identified in *A. baumannii* as reducing imipenem susceptibility, AdeABC [10], AdeFGH [11], and AdeIJK [12] are resistance-nodulation-division (RND) family pumps that have been associated with resistance to aminoglycosides, β-lactams, fluoroquinolones, tetracyclines, tigecycline, macrolides, chloramphenicol, and trimethoprim and increased transcription of three resistance-nodulation-cell division (RND) efflux pumps, has been related to carbapenem resistance in *A. baumannii* (CRAB) [8].

To date, various efflux pump inhibitors (EPIs) have been tested on *A. baumannii* such as Carbonyl cyanide m-chlorophenyl hydrazone (CCCP), Phenylalanine-arginine β-naphthylamide (PAβN), reserpine, omeprazole, 1-(1-Naphthylmethyl)-piperazine (NMP), and verapamil. Some of them had a remarkable effect when paired with antibiotics, while others had a limited effect [13–15]. To counteract the activity of efflux and reverse imipenem resistance, other efflux pump inhibitors (EPIs) have been developed, and maybe strong alternative adjuvant therapy instead of Carbonyl cyanide m-chlorophenyl hydrazone (CCCP), which has shown good activity but with toxicity [13].

Over a decade, antimicrobial inhibitory activity against a wide variety of pathogens has been documented by plants or plant-based compounds [16]. Because of their impact, the combination of essential oils and antibiotics has been recommended due to their effects by enhancing the antibiotic activity and reducing their toxicity, which is recorded as promising synergy action against Gram-negative bacteria in particular, cinnamon, thymol, clove, and caraway and so on that was supported by previous studies [17–21].

Therefore, we detected efflux pump genes in *Acinetobacter* species and their correlation to multidrug resistance, in particular, carbapenems-resistant *Acinetobacter baumannii* (CRAB) strains, and to use different plant-derived inhibitors and chemicals for restoring antibiotic susceptibility, especially of imipenem resistant in *A. baumannii.*

## Material and methods

### Study design

As part of their routine work, five clinical microbiological laboratories in Cairo, Egypt's El-Demerdash, Abo-El-Reesh for Children, El-Kasr Al-Ainy, and Red Crescent hospitals sequentially recovered 150 bacterial isolates thought to be *Acinetobacter baumannii* over the course of a year (December 2017–December 2018). Clinical specimens from the urinary tract, respiratory system, circulation, and wounds of hospitalized patients in the critical care unit yielded isolates. In our Microbiology Lab., *A. baumannii* isolates were identified presumptively through microscopic inspection and biochemical testing, including Gram staining, oxidase testing, catalase testing, motility testing, citrate utilization testing, oxidative/fermentative glucose (O/F) testing, growth capability at 44°C [22]. The *bla*_*OXA-51-like*_ beta-lactamase gene specific to *A. baumannii* was then found using PCR amplification (primer given in Table S1), by the prior methodology, to validate the identity of all isolates [23]. The reference strain, *A. baumannii* 17978, was used as a control throughout the study.

### Testing for antimicrobial susceptibility

Using the conventional disk diffusion technique and an interpretation of the breakpoint criteria defined according to Clinical and Laboratory Standards Institute (CLSI) recommendations, the antibiotic resistance profile of 200 *A. baumannii* isolates was determined [24]. The antibiotics used in this study; gentamicin (GM, 10μg), tobramycin (TOB, 10μg), amikacin (AK, 30μg), amoxicillin/clavulanate (AMC, 10/10μg), ampicillin/sulbactam (SAM, 10/10μg), piperacillin (PIP, 100μg), cefepime (FEP, 30μg), cefotaxime (CTX, 30μg), ceftazidime (CAZ, 30μg), ceftriaxone (CRO, 30μg), imipenem (IPM, 10μg), ciprofloxacin (CIP, 5μg), ofloxacin (OFX, 10μg), colistin sulfate (10μg), and Trimethoprim/Sulfamethoxazole (SXT, 1.25/23.75μg).

All the disks were bought from Oxoid in the USA. A bacterial suspension equal to 0.5 McFarland (1.5 x 10^5^ CFU (colony forming unit)/ml) was added to cation-adjusted Mueller Hinton Agar (MHA) plates from Merck (Germany), and these plates were then incubated at 37°C for 18–24 hours.

### Minimum inhibitory and bactericidal concentrations (MIC & MBC)

A variety of efflux pump inhibitors including, chemical drugs, trimethoprim, and proton pump inhibitors such as omeprazole, esomeprazole, and pantoprazole (GSK pharmaceuticals, Egypt), and essential oils, such as Cinnamon oil (*Cinnamomum verum*), Clove oil (*Syzygium aromaticum*), Thyme oil (*Thymus vulgaris*), and Caraway oil (*Carumcarvi*) (Al-Andalos comp. Egypt) compared with a standard efflux pump inhibitor, carbonyl cyanide 3-chlorophenylhydrazone (CCCP, Sigma Aldrich, USA) were examined their MIC against the selected thirty-seven clinical CRAB strains using the resazurin microtiter plate assay CLSI 2014 [24, 25], which used as the redox indicator resazurin that changed color from blue to pink in the presence of viable cells. The minimum bactericidal concentration (MBC) is defined as the lowest concentration of the inhibitor with no bacterial growth by plating 5 μL from each well on Trypticase soya agar medium after incubation at 37°C for 24 h compared with the positive control (broth media).

A stock solution of CCCP, trimethoprim, and omeprazole was prepared at 100mg/mL in 1% DMSO, a similar concentration of pantoprazole and esomeprazole were solubilized in ddH2O [26], and stock solutions of essential oils were prepared at 4% (v/v) in 5% tween 60 to emulsify the oils without exerting any antibacterial activity on the CRAB strains [27]. Serial two-fold dilution was used to calculate MICs against the tested strains, CCCP (4-1024 mg/L), chemical drugs (10-50 mg/L), and essential oils (0.03-4 % v/v).

### Checkerboard combination between imipenem and efflux pump inhibitors on CRAB strains using broth microdilution

Imipenem MIC concentration used was ¼ MIC obtained from minimum inhibitory concentration. The MIC was determined as the concentration at which there was no color change following 4 h incubation of the overnight cells with 0.015% resazurin dye (Sigma Aldrich, USA). Fold decreases in MICs of imipenem were calculated using Eq. 1 [28] using FICI (Fractional Inhibitory Concentration Index).

1 $$\mathcal{FICI}=\mathcal{FICA}+\mathcal{FICB}$$

Where:

$$FICA = MIC{combination}/MIC{alone}$$

$$FICB = MIC{combination}/MIC{alone}$$

### Time-kill assays

To confirm the bactericidal effect of combined pump inhibitors and imipenem, the time-kill analysis was performed for two clinical carbapenem-resistant *A. baumannii* strains (CRAB15 and CRAB 99). Imipenem, and selected EPIs (CCCP, trimethoprim, and cinnamon oil) were tested alone and in combinations at concentrations depending on MIC determined from microbroth chequerboard testing and showed synergistic action at time intervals 0, 2, 4, 16, and 24 h. The percentage of dead cells is calculated relative to the growth control by determining the total bacterial log10 CFU/ml of living cells (CFU/ml) of each tube using the agar plate count method [29] in CA-MHB supplemented with imipenem. A ≥ 2 log10 drop in CFU/mL with the medication combination relative to its most active ingredient following 24 h and a ≥ 2 log10 decline in the CFU/mL under the initial inoculum were necessary for the term "synergy" to be understood. However, if the CFU/mL increased by 2 log 10 or decreased by ≥ 2 log10, the medication combination was interpreted as "antagonistic," and a < 2 log10 shift in CFU/ml was interpreted as "no interaction".

### Gas chromatography-mass spectrometry (GC-MS) analysis for cinnamon and thyme oils

The analysis for the two most effective essential oils, cinnamon, and thyme oils was carried out in the Agricultural Research Center (ARC), Egypt, to determine their chemical composition, using a GC-MS (Agilent Technologies 7890A) interfaced with a polar Agilent HP-5ms (5%-phenyl methyl poly siloxane) capillary column 30m x 0.25 mmi.d. and 0.25μm film thickness [30].

### Amplification of carbapenem resistance efflux pump genes

Amplification of genes encoding efflux pumps, *adeB, adeJ, adeK, adeC* (AdeB, AdeC, AdeJ, and AdeK) of RND family and housekeeping *rpoB* genes was carried out using the genomic DNA of 37 CRAB strains by PCR to detect which supposed to be responsible for carbapenem resistance in *Acinetobacter baumannii* strains. The primer sequences used for RT-qPCR are listed in Table S1.

#### RNA extraction and cDNA synthesis

Using RT-qPCR, the overexpression of four different genes in two CRAB strains (which showed synergistic activity with EPI) was identified. The bacterial strains were cultivated overnight at 37°C on CA-MHA plates with imipenem at sub-inhibitory concentrations (8 and 128 g/mL). Total RNA of cells was extracted using a Qiagen RNeasy Mini Kit (Qiagen, Germany, GmbH) from late log-phase cultures of two different CRAB strains (ACN15 and ACN99), and the RT-qPCR reaction was carried out according to the manufacturer's instructions using Qiagen one-step RT-PCR Kit (Thermo-fisher, USA). Once RNA was clean and free of DNA, cDNA was made from template RNA (0.5 g) using a reverse transcriptase kit from Thermo Fisher in the United States using the kit's oligo primers. 20°C was used to store the cDNA.

#### Relative expression of efflux pump genes by quantitative real-time-polymerase chain reaction (RT-PCR) using efflux pump inhibitors (EPI)

Efflux pump inhibitors (EPI) were employed to detect the overexpression of the efflux pump genes *adeB, adeC, adeJ,* and *adeK* in *A. baumannii* strains, and real-time RT-qPCR (Applied Biosystems) was utilized to assess the gene transcription. The primer sequences used for RT-qPCR are listed in Table S1. The housekeeping gene ropB (626 bp/base pair), which is utilized as an internal control for RT-PCR, was used. The SYBR Green RT-PCR mixture was made up of 500 ng of the cDNA, 0.25 mM of each primer, reaction mixture 2 of the QuantiTect SYBR Green PCR kit, and distilled water in a 25 L volume. The reaction was carried out in two initial steps of 50 °C for 30 min (reverse transcription) and 94 °C for 15 min to activate the Taq polymerase, then 40 cycles of denaturation at 94 °C for 15 sec., annealing at 53 °C for 30 sec. (*adeB and adeC*), at 46 °C for 30 sec. (*adeJ, and adeK*), and extension at 72 °C for 40 sec [31]. By standardizing the expression of the *ropB* gene by the following equation, the gene expressions were estimated utilizing the compared threshold cycle (CT) approach:

$$\textrm{RQ}=2^{-\triangle\triangle\mathit{CT}},$$

Where CT is the value corresponding to the crossing point of the amplification curve with the threshold; ΔCT = target CT or calibrator CT – endogenous CT; and ΔΔCT = target ΔCT – calibrator CT.

### Statistical analysis

Data analysis was carried out by GraphPad Prism v8.4.3 Software using the student’s t-test. In all analyses, a two-tailed probability, *P-value* <0.05 was considered statistically significant and highly significant when ****P <0:0001. The experiments were performed in triplicate.

## Results

### Antimicrobial susceptibility testing

**Fig. 1:**
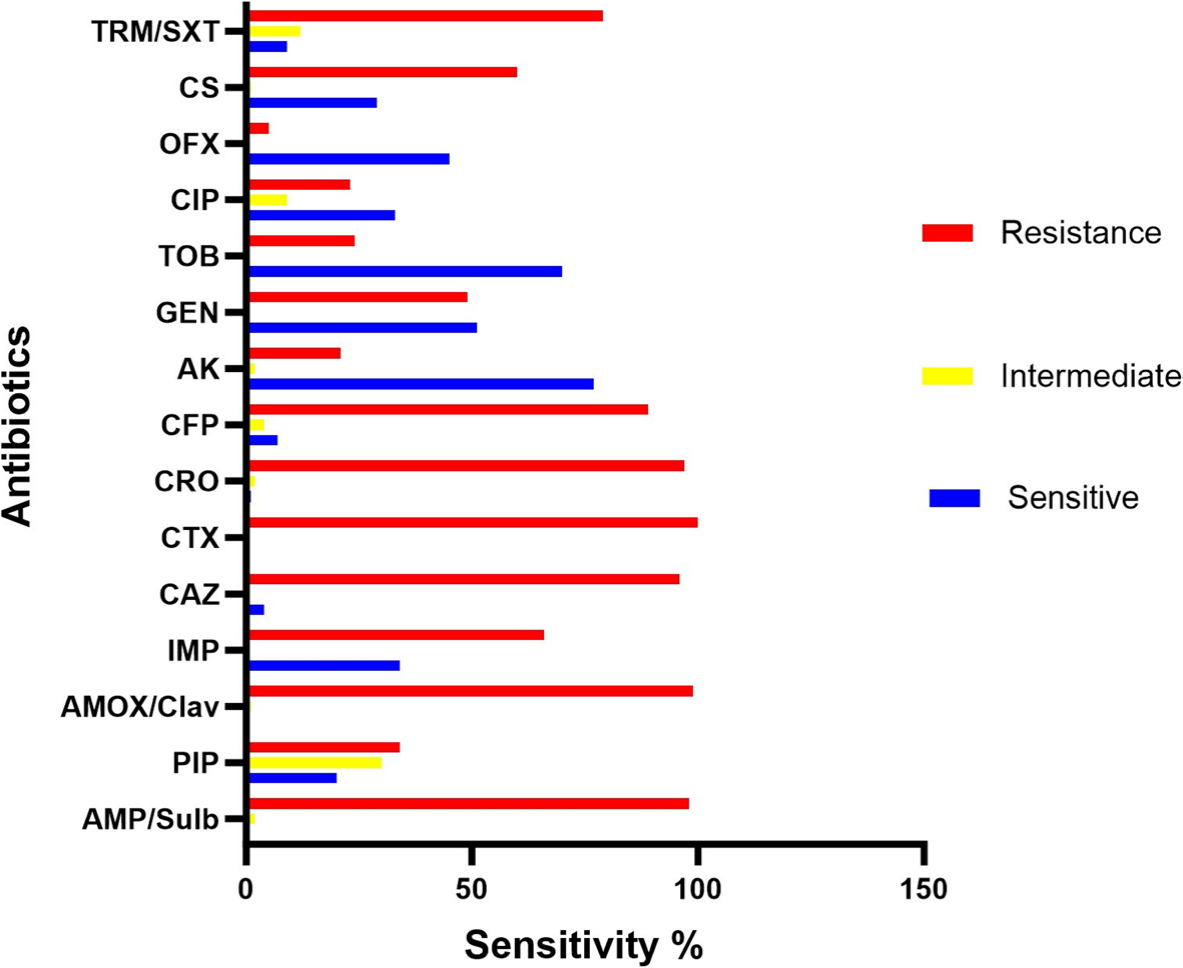
Antimicrobial susceptibility of *A. baumannii* isolates. SAM: ampicillin/sulbactam, PIP: piperacillin, AMC: amoxicillin/clavulanate, IMP: imipenem, CRO: ceftriaxone, CTX: cefotaxime, FEP: cefepime, Ak: amikacin, GN: gentamicin, TOB: tobramycin, CIP: ciprofloxacin, OFX: ofloxacin, CT: colistin sulphate, SXT: Trimethoprim/Sulfamethoxazole

Over one year of the bacterial samples collection, we enrolled 100 out of 120 clinical isolates meeting identification criteria in this study as *A. baumannii* based on morphological, phenotypic, and molecular characteristics by detection of *bla*_*OXA-51*- like_ carbapenemase gene as an intrinsic gene (353bp). *A. baumannii* classification according to sensitivity results to all antibiotics is given in Fig. 1, indicates that all clinical *A*. *baumannii* isolates were multidrug-resistant (MDR) isolates with the high resistant rate observed against CTX (100%), AMC (99%), SAM (98%), CRO (97%), CAZ (96%) and FEP (89%). However, 79% of clinical isolates expressed resistance to SXT and 66% to IMP using disk diffusion method. However, all 37 strains classified as imipenem resistant (IMP-R) isolates, fell in the IMP-R category according to CLSI guidelines using broth microdilution. These 37*A.baumannii* isolates are also resistant to almost all antibiotics used in this study. All the 37 IMP-R isolates had MICs (MIC_50_) median value of 64 μg/ml (8-1024μg/ml) as demonstrated in Table 1.

**Table 1:** Minimum inhibitory concentrations (MICs) of the imipenem after treatment with efflux pump inhibitors against clinical CRAB strains

Strains	Treatment							
	MICs (mg/L)							
	IMP^a^	+TMP^b^	+OMP^c^	+CCCP^d^	+Cinnamon oil	+Thyme oil	+Clove oil	+Carraway oil
**ACN 91**	**32**	8	8	4	4	8	8	32
**ACN 92**	**16**	8	2	4	4	8	4	4
**ACN 93**	**8**	4	2	2	4	4	4	4
**ACN 94**	**16**	4	8	2	4	4	8	8
**ACN 95**	**16**	4	4	2	2	8	8	8
**ACN 96**	**16**	4	4	2	2	8	8	8
**ACN 97**	**128**	8	16	8	32	16	16	16
**ACN 98**	**64**	8	4	8	2	16	16	8
**ACN 99**	**256**	8	128	16	16	16	64	64
**ACN 100**	**1024**	64	64	64	64	64	256	1024
**ACN 70**	**64**	16	8	4	8	16	8	16
**ACN 71**	**64**	8	16	4	8	16	8	16
**ACN 72**	**64**	8	8	4	32	16	8	16
**ACN 73**	**64**	16	16	8	32	16	8	16
**ACN 74**	**32**	8	16	4	4	8	8	32
**ACN 75**	**32**	8	4	2	2	8	8	32
**ACN 76**	**32**	4	8	4	8	16	8	32
**ACN 77**	**32**	8	8	4	2	16	8	32
**ACN 78**	**64**	16	8	4	8	8	16	16
**ACN 79**	**64**	8	16	8	8	16	16	8
**ACN 80**	**64**	8	16	8	8	NA	NA	NA
**ACN 81**	**32**	8	8	2	8	NA	NA	NA
**ACN 82**	**128**	32	64	16	2	NA	NA	NA
**ACN 83**	**64**	16	16	8	2	NA	NA	NA
**ACN 84**	**128**	16	16	16	32	NA	NA	NA
**ACN 23**	**256**	64	16	16	16	NA	NA	NA
**ACN 16**	**128**	16	16	16	16	NA	NA	NA
**ACN 47**	**64**	8	32	8	2	NA	NA	NA
**ACN 65**	**32**	4	8	8	2	NA	NA	NA
**ACN 54**	**512**	128	64	8	16	NA	NA	NA
**ACN 13**	**512**	32	64	16	16	NA	NA	NA
**ACN 43**	**16**	2	4	4	2	NA	NA	NA
**ACN 17**	**256**	8	32	32	16	NA	NA	NA
**ACN 14**	**128**	32	32	4	8	NA	NA	NA
**ACN 59**	**128**	32	64	4	8	NA	NA	NA
**ACN 1**	**256**	32	16	16	16	NA	NA	NA
**ACN 15**	**16**	2	4	2	2	NA	NA	NA

^a^*IMP* Imipenem, ^b^*TMP* Trimethoprim, ^c^*OMP* Omeprazole, ^d^*CCCP* Carbonyl cyanide 3-chlorophenylhydrazone, *NA* No action

### Challenge of efflux pump inhibitors (EPI) for reduction MICs of imipenem-resistant (IMP-R) strains

The MICs and MBC of efflux pump inhibitors were tested alone (Table S2) and in combination with imipenem as mentioned in Table 1, against 37-IMP-R strains to inhibit the efflux potential of CRAB strains. All strains grew in the presence of CCCP at the concentration up to 1024 mg/L in DMSO without imipenem, confirming that CCCP alone does not affect this concentration on *A.baumannii*; whereas up to 50 mg/L of pantoprazole and esomeprazole, there is no effect observed against these strains, however, omeprazole showed effect at 50 mg/L; however, trimethoprim showed a variable effect range from 5 mg/L (3 CRAB strains), 15 mg/L (15 strains), 25 mg/L (20 strains). Moreover, cinnamon oils had an inhibitory effect alone in the range from 0.25-2% against CRAB strains, also thyme oil had an inhibitory effect but only against 3 strains; however, all other tested essential oils had no effect up to >4%.

A simple phenotypic marker for the efflux resistance mechanism is the CCCP addition to imipenem showed a reverse in imipenem MIC that confirms the presence of imipenem resistance mechanism through fold change in MIC (Table 2). Out of essential oils used as inhibitors, cinnamon oil was the most active inhibitor recorded synergistic action according to FIC index against all tested clinical CRAB strains with MIC fold change ≥ 2, conferring the efflux pump prevalence as a resistance mechanism in CRAB strains. Only three isolates had fold change <2 of MIC _IMP_, but these isolates had low MIC _IMP_ (8 and 64 mg/L) and acceding to FIC index it showed synergy action (Table 3), however, five strains showed high 5-fold change in MIC _IMP_ and one strain had 6-fold change that led to reverse imipenem resistance MIC to 2 mg/L.

**Table 2:** Imipenem MIC Fold change after treatment with Efflux Pump Inhibitors against clinical CRAB strains

Treatments	No. (%) of CRAB strains		
	≥ 2-fold	≥ 4-fold	≥ 6-fold
^**a**^**IMP+TMP**^**b**^ (*n*=37)	35 (94.5)	4 (10.8)	-
**IMP+OMP**^**c**^ (*n*=37)	31 (83.7)	4 (10.8)	-
**IMP+CCCP** (*n*=37)	8 (21.6)	-	-
**IMP+Cinnamon** (*n*=37)	34 (91.8)	15 (40.5)	1 (2.7)
**IMP+Thyme** (*n*=20)	13 (65)	2 (10)	-
**IMP+Clove** (*n*=20)	16 (80)	-	-
**IMP+Carraway** (*n*=20)	10 (50)	-	-

^a^*IMP* Imipenem, ^b^*TMP* Trimethoprim, ^c^*OMP* Omeprazole

**Table 3:** Combination action of efflux pump inhibitors after treatment against clinical CRAB strains

Treatments	Number of strains (%)		
	S^a^	Ad^b^	Ag^c^
**IMP**^**d**^**+TMP**^**e**^ (*n*=37)	16 (43.2)	19 (51.3)	2 (5.4)
**IMP+OMP**^f^ (*n*=37)	21 (56.7)	15 (40.5)	1 (2.7)
**IMP+Cinnamon** (*n*=37)	37 (100)	0	0
**IMP+Thyme** (*n*=20)	20 (100)	0	0
**IMP+Clove** (*n*=20)	20 (100)	0	0
**IMP+Carraway** (*n*=20)	14 (70)	0	0

^a^*S* Synergistic, ^b^*Ad* Additive, ^c^*Ag* Antagonistic, ^d^*IMP* Imipenem, ^e^*TMP* Trimethoprim, ^f^*OMP* Omeprazole

Furthermore, the addition of thyme oil to imipenem reported two strains with a 4-fold change in MIC of imipenem that reversed from 256 and 1024 mg/L to 16 and 64 mg/L at 0.25 and 0.5% of oil, respectively as shown in Table 1.

However, MIC _IMP_ reported a 3-fold change against five strains with reversing imipenem resistance to 16 and 8 mg/L, with the addition of clove oil, and in the case of caraway oil, one strain achieved a 3-fold change in MIC _IMP_ (MIC 16mg/L). Synergistic effects of imipenem and thyme, clove, and caraway were observed against 20 and 14 CRAB strains, respectively. However, with the addition of all other plant-based inhibitors, no action has been registered (Table 3).

Out of 37 combinations of inhibitors, trimethoprim and omeprazole have synergistic action against 16 and 21 tested clinical CRAB strains, respectively with ≥ a 2-fold change in MIC _IMP_ (Table 2 and Table 3). Only one isolate with the addition of trimethoprim had a 5-fold change in MIC _IMP_ and reversing imipenem resistance from 256 to 8 mg/L. Two strains had a 4-fold change in MIC_IMP_ with reversed imipenem resistance to 4 and 16 mg/L. With the addition of efflux inhibitors, pantoprazole, and esomeprazole, there is no change in imipenem MICs up to 50 mg/L concentrations against all isolates. The addition of trimethoprim and omeprazole to imipenem recorded additive (19 and 14) and antagonism action (2 and 1, respectively) against some strains have been reported in this study, and with the addition of essential oil to imipenem, no additive or antagonism reaction have been reported Table 3.

### Time-kill kinetics

**Fig. 2:**
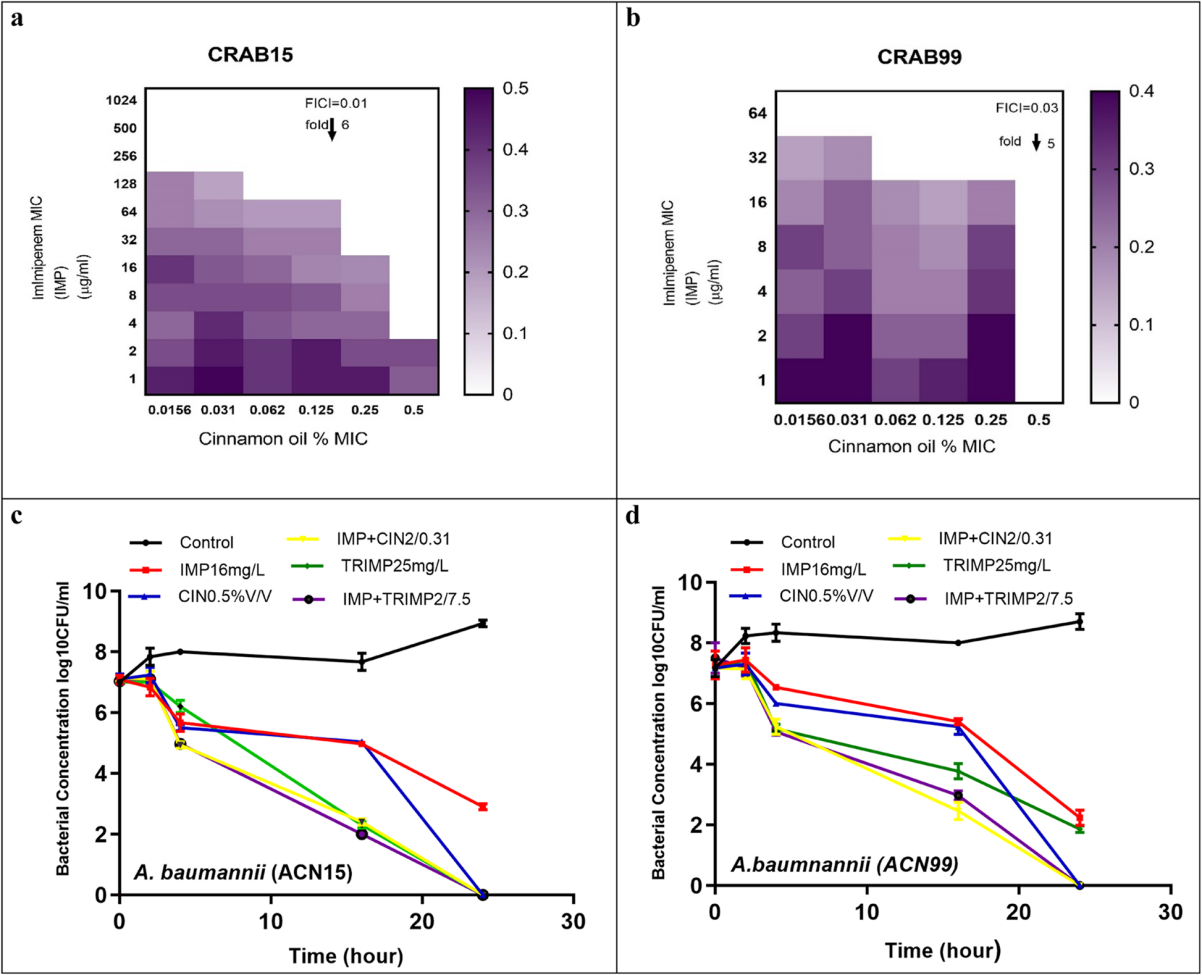
Effect of treatment with EPI combined with IMP against two CRAb study strains (strains ACN15, and ACN99), where, (**a**, **b**): synergistic activity between IMP and cinnamene oil against CRAB isolates by checkerboard assay, Dark color represent higher density, and (**c**, **d**): bactericidal assays using imipenem combined with trimethoprim and cinnamon oil. y axis, log_10_ CFU/ml; x axis, time in hours; 2a, CRAB strain ACN15, 2b, CRAB strain CAN 99, black line: control strain without treatment, red: Imipenem alone at 16mg/L MIC; blue line: cinnamon oil alone at 0.5% (v/v), yellow line: Imipenem plus cinnamon oil at 2 mg/L/0.31% (v/v); green line; trimethoprim alone at 25 m g/L; violet line: synergy assay using imipenem plus trimethoprim at 2 mg/L/7.5mg/L

To confirm the impact of EPIs, to show synergistic activity (Fig. 2a and b), on imipenem activity, we performed the time-kill assays of imipenem for two selected CRAB strains (ACN15, ACN99), in the presence or absence of cinnamon oil and trimethoprim at two different concentrations 16 and 8 mg/L, respectively. These data show that *in vitro* susceptibility of imipenem can be influenced by the addition of EPIs. The killing curve of ACN99 isolate represented the association of imipenem + trimethoprim as a bactericidal activity with bacterial count reduction to 5 log_10_ after 4 h and complete killing after 24 h. Similar activity was noticed for imipenem + cinnamon oil after 24 h (Fig. 2d)

The killing curve of imipenem in conjunction with trimethoprim and cinnamon oil against ACN15 isolate at a concentration that displayed the synergistic activity achieved a bactericidal activity after 24 h (Fig. 2c) with reversed imipenem resistance to 2 mg/L for both reactions, although regrowth was observed in the presence of imipenem alone. The study showed the effectiveness of imipenem against strains increased in combination with trimethoprim and with cinnamon oil.

### GC-MS analysis of cinnamon and thyme oils

Analysis of cinnamon and thyme oil using GC-MS represented the components that affect oil’s effectiveness and in combination therapy, longifolene (45.08%) was the predominant one followed by coumarin (33.90%), linalool (18.54%), isoeugenol (15.29%), α-pinene (12.0%), cinnamyl alcohol (10.84%), cinnamaldehyde (6.69%) and low concentration of α-Terpinene (6.38%) and thyme oil contains thymol (51.8%) as a major component, *p*-cymene (29.05%), and linalool (5.24%) Table S3.

### Amplification and regulation of efflux gene expression after challenge with EPIs

**Fig. 3:**
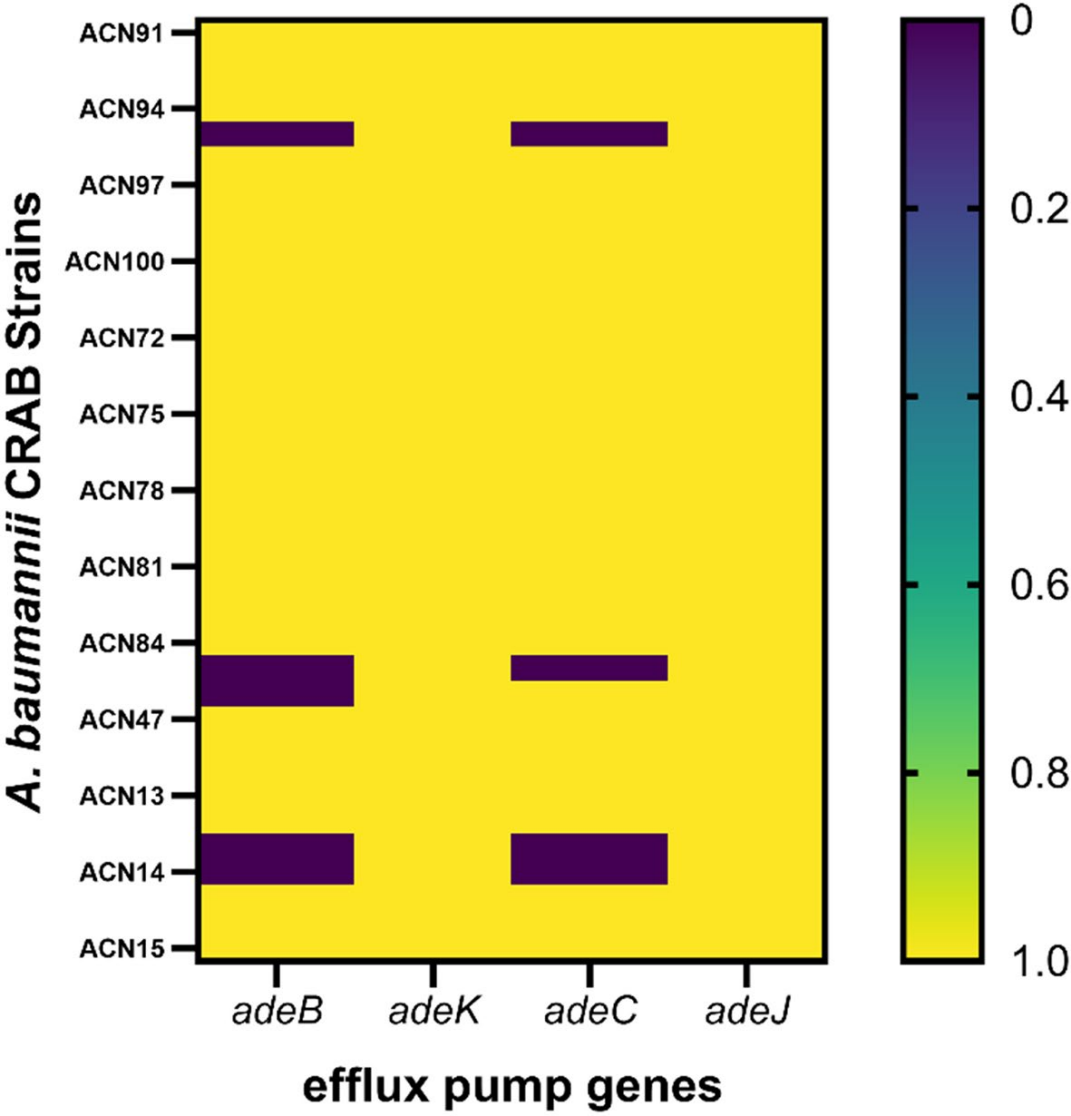
Amplification of efflux pump genes in thirty-seven CRAB strains

**Fig. 4:**
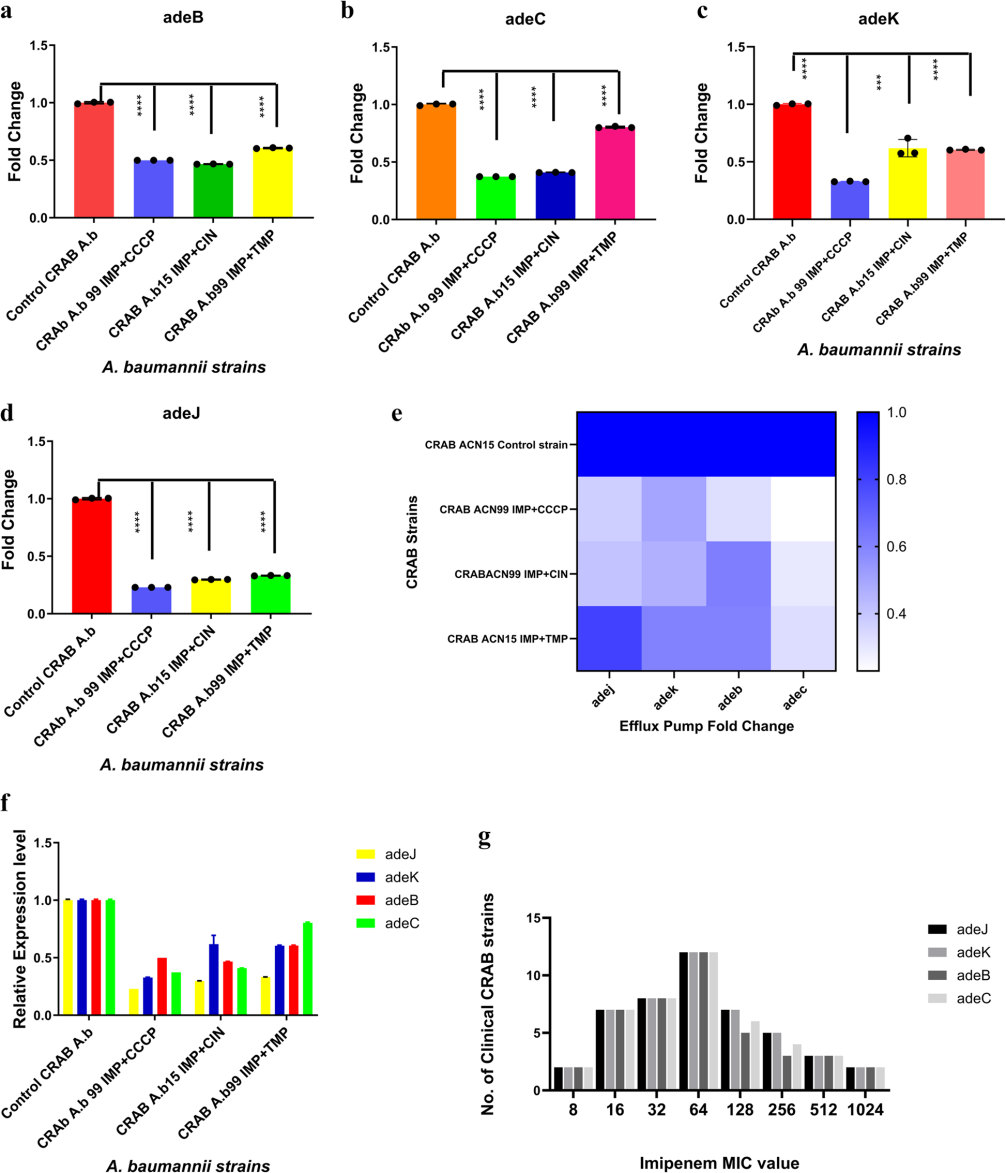
Relative expression level of efflux pump genes with combination treatment of imipenem with cinnamon oil, trimethoprim and CCCP against two clinical CRAB strains (ACN15, and ACN99). RT-qPCR test was done to assess the effect of the treatments on AdeABC and AdeJIK in *A. baumannii strains*. Relative mRNA levels of target efflux pumps genes; **a**. *adeJ,* **b**. *adeK*, **c**. *adeB,* and **d**. *adeC* were normalized to the geometric mean of reference genes (*rpoB*). The vertical bars represent mean ± S.D of triplicate independent experiments (n = 3). Data were analyzed using t-student test (****p <0.0001). *A. baumannii* CRAB ACN15, and ACN99 strains, and *A. baumannii* strain (ACN 15, control strain) were grown at a sub-inhibitory concentration of IMP (8 and 128 μg/mL) for control strain and EPI (cinnamon oil, Trimethoprim, and CCCP) combined with IMP overnight at 37^°^C on CA- MHA plates, **e**. Heatmap graph represents fold change in relative gene expression of *adeJ, adeB, adeC and adeK* genes of CRAB strains (ACN15, and ACN99) after combination treatments comparing with *A. baumannii* strain (ACN15, control strain), **f**. Bimodal figure for to dissemination of amplified efflux pump genes associated with the AdeABC and AdeIJK system in two selected isolates after treatment with EPI, and **g.** Bimodal figure for distribution of imipenem MICs in 37 clinical CRAb strains classified according to dissemination of amplified efflux pump genes associated with the AdeABC and AdeIJK system

Among the thirty-seven clinical CRAB strains, the *adeJ*, *adeK, adeB*, and *adeC* genes were detected in the tested strains as follows: 100% (37/37), 100% (37/37), 86% (32/37), and 94.5% (35/37)*,* respectively (Fig. 3). Figure S1 shows the electrophoretogram of the PCR amplification of the efflux pump genes in the tested CRAB strains. Furthermore, the efflux pump genes have analyzed the expression of those genes using RT-qPCR to study the combined effect on two CRAB strains (ACN15 and ACN99) before and after the addition of efflux pump inhibitors (Fig. 4). Each relative expression value was the mean of 3 replications The mRNA levels of *adeJ, adeK, adeC,* and *adeB* genes in ACN99 strain were significantly downregulated (****P<0.0001) after the addition of imipenem combined with cinnamon oil also imipenem and CCCP than those in control strain, and the mRNA levels of *adeJ, adeK, adeC,* and *adeB* genes in ACN99 strain were significantly decreased (****P<0.0001) after addition of imipenem combined with CCCP when compared with control CRAB strain (ACN15) without treatment. In addition, the mRNA levels of *adeJ, adeK, adeC,* and *adeB* genes in ACN15 strain were significantly decreased (****P<0.0001) after we added imipenem combined with trimethoprim when compared with control as illustrated in Fig. 4a-d. Heatmap showed the downregulation of four efflux pump genes Fig. 4e.

Data in Fig. 4f, represented the bimodal distribution of the amplified efflux genes and their correlation to MIC values, most of the thirty-seven strains, 32(86.48%) have high MIC values yielded a positive result, except *adeB*, and *adeC* that was absent in 5 (15.62%) and 2 (5.4%) strains respectively, and showed low MIC.

Based on our results, the combination treatment of imipenem and cinnamon oil showed a stronger inhibitory effect on the regulation of four RND efflux pump genes than the imipenem and trimethoprim combination treatment in comparison with CCCP combination therapy as a standard. Imipenem-cinnamon oil combination is considered a treatment option block resistance gene, the most downregulated gene was *adeJ* gene followed by *adeB* gene then *adeK* gene, and finally, *adeC* gene as was represented in the heatmap (Fig. 4g).

## Discussion

Infections caused by *A. baumannii* has been reported worldwide and the current regimes used for treatment registered their failure due to the spread of multiple resistance genes prevailing in different mechanism of resistance; therefore, the abolishment of those genes is considered a paramount scope in our investigation. Efflux pump resistance genes belonging to RND family, which are classified in *A. baumannii* have been shown to play an important role in *A. baumannii* resistance to imipenem*,* now representing a candidate target for the development of antibiotic resistance therapy.

The chromosomally identified gene in this species, *bla*_*OXA-51*-like_ carbapenemase, was used in our investigation to determine which of the 100 bacterial strains collected from Egyptian hospitals belonged to *A. baumannii*. Ideally, such a marker should distinguish this species from other species in *Acinetobacter* genus and is a straightforward, trustworthy, and dependent genetic marker [23, 32, 33].

*A. baumannii* strains showed high resistance rates to almost all antibiotics with special concern for imipenem, which was detected in 66% of strains. Earlier Egyptian studies reported the prevalence of imipenem resistance and the rate was high (50-70%), while others were coming up to 100% proved through a wide MICs range (16-1024 mg/L) in 37 CRAB strains, and such agreement between our data and the quoted one may suggest the development of imipenem resistance with years and it needs necessary continuous surveillance [34–37] in healthcare facilities especially intensive care units. Similarly, imipenem resistance recorded high resistance dissemination globally, this problem was recently documented in surveys carried out in Iran (85%), Saudi Arabia (100%) [38], and Algeria (80.91%) [39].

On the other hand, amikacin was found to be an effective drug against *A. baumannii* strains; 21 % of strains were resistant to amikacin and this rate was lower than those obtained in previous studies as following 45%, 76.9%, 90%, 81.5%, and 33%, respectively [34, 36].

Although colistin sulfate is the last resort for the treatment of *A. baumannii* infections, colistin resistance has been reported in 60% of strains in our study, and this higher rate is recently documented previously [37, 40, 41]. Correspondingly, some studies revealed that colistin retains its effectiveness against *A. baumannii* in Egypt (4.5%, and 7.4%) by El-Masry and Masry and Elsherbeny et al., respectively [42, 43].

The role of efflux in imipenem resistance has been reported in several studies and considered as a main mechanism alongside the enzyme degradation, in our study, we proposed that a reversal pattern on imipenem resistance with the addition of CCCP in 26 of 37 strains, supporting the fact that blocking the efflux pump is crucial for the imipenem resistance even though multi-drug resistance [41, 44].

Although imipenem is commonly used in the treatment of *A. baumannii* infection, a single administration of this drug becomes infective because of the development of carbapenem-resistant *A. baumannii* isolates. In addition, using alternative therapeutic regimes for CRAB strains such as polymyxins, tigecycline, and aminoglycosides led to failure due to increasing resistance rates and pharmacokinetic properties [45]. The limited therapeutic options, as well as the long time and high cost required for the development of novel drugs have prompted increased interest in seeking efficient alternative approaches for the eradication of drug-resistant bacterial agents based on existing drugs as an attractive alternative combination therapy, Nickel nanoparticles (NiNPs) [46] and efflux pumps inhibitors demonstrated substantial effects against drug-resistant bacterial agents. One of the promising challenges to manipulating the resistance problem is the blocking of efflux pumps using inhibitors to open a broad chance to adjuvant therapy that revives antibiotic effectiveness [47]. In our study, several compounds of different natures were used as inhibitors to narrow the imipenem resistance pattern. Among 13 inhibitors used, cinnamon oil, thyme oil, caraway oil, clove oil, trimethoprim, and omeprazole were the effective inhibitors used that proved through the reduction of MICs of imipenem with up to 6-fold change. The results are congruent with previous reports [19, 20].

The inhibitory effect of oil when combined with antibiotics attributed to their components, *p*-cymene linalool, thymol, trans-cinnamaldehyde, and eugenol, where they act permeabilization of the bacterial membrane through disruption of the negatively charged outer membrane and resulting in increased penetration inside the cell. Cinnamon oil was used as a natural preservative and a flavoring substance that is not harmful when consumed, hence we suggest that it can be applied clinically, especially Becerril *et al.* study proved that *A. baumannii* inhibited by cinnamon oil even after 50 sequential passages in the subinhibitory concentrations of cinnamon oil [21].

The results agreed with the results of Miladi [48] who conducted a study to evaluate the anti-efflux activities of five EOs components (eugenol, carvacrol, thymol, p-cymene, and γ-terpinene) alone or in combination with tetracycline against *S. aureus* ATCC 25923. Our results are also in line with those of Abdelatt and his co-workes who showed that there was a significant decrease in the expression levels of MexA and MexB genes of *P. aeruginosa* isolates treated with cinnamon oil when compared to the non-treated ones [49].

On the other hand, pyrimidine ring in trimethoprim explains the synergistic combination with imipenem and ciprofloxacin that gave this synergy. Trimethoprim has a pyrimidine ring, that seems to be a common component of several EPIs, including Pa-N, and an aromatic ring connected by a link to a basic nitrogen-containing moiety [50, 51], but omeprazole is designated as a proton pump inhibitor, its effect provided from its ability to block H^+^ considered as a potential inhibitor of EP families using the H^+^ gradient (drug/H+ antiporters) to eject antimicrobial drugs for the cytosol such as MFS, SMR, and RND efflux pump families in line with the recent publication [20], or maybe through inactivation of H^+^, K^+^ ATPase in bacterial cells, which affects the uptake of drug.

Interestingly, the time-kill study showed the association of both cinnamon and trimethoprim with imipenem was bactericidal activity against two CRAB strains (ACN15 and ACN99) after 24 h, supported by other data on *A. baumannii* using cinnamon oil or its components [18, 52, 53]. The primary step for strains to become fully resistant is the overexpression of chromosomal efflux pump genes [8]. Therefore, the manipulation of these pumps to provide a feasible alternative therapeutic regimen is the principal target of this study. Our investigation described the presence of *adeK, adeJ, adeB, and adeC* proved the decreased susceptibility to imipenem and suggests an important increase of this carbapenems circulation, which is constantly found in *A. baumannii* in Egypt, which also, might indicate the up-regulated of two families, AdeABC, and AdeJKL.The loss of *adeC*, which is assumed to encode a membrane on the outside porin but was only found in 2 strains, despite the presence of *adeB*, may be attributed to the reality that this porin is not required for the efflux activities [54]. To assess the involvement of efflux pump inhibitors in the downregulation of efflux pump genes, RT-qPCR is used as a transcriptome marker for using these inhibitors for the emergence of an alternative therapy against these pathogens. Expression of AdeJKL family genes, *adeJ,* and *adeK* was the most predominant family and the most down-regulated genes with the addition of both cinnamon oil and trimethoprim when compared with AdeABC family genes, *adeA,* and *adeB*, suggesting that the AdeIJK complex may contribute carbapenem resistance in *Acinetobacter* alongside AdeABC; consistent with the observation of Karumathil et al., Higgins et al., and Damier-Piolle et al. [9, 12, 18].

## Conclusion

We concluded that the MDR *A. baumannii* circulation in Egyptian hospitals underlined the importance of regimes in limiting their emergence. The study findings highlight the association between carbapenem resistance in *A. baumannii* and AdeABC, and AdeJKL overexpression as observed by the addition of efflux pump inhibitors that reversed the carbapenem resistance. Using the combined form of imipenem and cinnamon oil, imipenem and trimethoprim were the effective combinations against all strains tested. Furthermore, the genes whose expression was altered upon exposure to a sub-MIC concentration of imipenem were affected by cinnamon addition rather than trimethoprim and this nominates imipenem plus cinnamon to be a cornerstone drug in combination therapy and subsequently, may reduce the imipenem dose and the adverse reaction.

### Supplementary Information

**Additional file 1.** Supplementary materials are associated with this article.

## Data Availability

The datasets used and/or analyzed during the current study are available from the corresponding authors on reasonable request.

## Abbreviations

bp: base pair
CCCP: Carbonyl cyanide m-chlorophenyl hydrazine
CFU: colony forming unit
CLSI: Clinical and Laboratory Standards Institute
CRAB: carbapenems-resistant *Acinetobacter baumannii*
CT: threshold cycle
EPI: Efflux pump inhibitor
ESKAPE: *Enterococcus faecium, Staphylococcus aureus, Klebsiella pneumoniae, A. baumannii, Pseudomonas aeruginosa, and Enterobacter spp*
FICI: Fractional Inhibitory concentration index
IMP-R: imipenem resistant
MBC: Minimum bactericidal concentrations
MIC: Minimum inhibitory concentrations
NMP: omeprazole, 1-(1-Naphthylmethyl)-piperazine
PaβN: Phenylalanine-arginine β-naphthylamide
RND: Resistance-nodulation-cell division
