# Supplementary material for: Regulation of overexpressed efflux pump encoding genes by cinnamon oil and trimethoprim to abolish carbapenem-resistant Acinetobacter baumannii clinical strains
Source: BMC Microbiol. 2024 Feb 8;24:52. doi: 10.1186/s12866-024-03194-8 (PMC10851603; doi:10.1186/s12866-024-03194-8)
Supplement: Supplementary file 1 — Additional file 1. Supplementary materials are associated with this article. [file 12866_2024_3194_MOESM1_ESM.docx]

**Table S1: Primers used in this study.**

| **Gene name** | **Sequence (5’-3’)** | | **Specificity** | **Size base pair (bp)** | **Reference** |
| --- | --- | --- | --- | --- | --- |
| *bla_OXA-51-like_* | F | TAATGCTTTGAT CGGCCTTG | *A, baumannii* detection gene | 353 | [22] |
|  | R | TGGATTGCACTTCATCTTGG |  |  |  |
| *rpoB* | F | ATGCCGCCTGAAAAAGTAAC | Housekeeping gene/Rt-qPCR | 154 | [23] |
|  | R | TCCGCACGTAAAGTAGGAAC |  |  |  |
| *adeB* | F | CTTGCATTTACGTGTGGTGT | Efflux pump genes/RT-qPCR | 168 |  |
|  | R | GCTTTTCTACTGCACCCAAA |  |  |  |
| *adeC* | F | TACACATGCGCATATTGGTG |  | 117 |  |
|  | R | CGTAAAATAACTATCCACTCC |  |  |  |
| *adeJ* | F | GGTCATTAATATCTTTGGC |  | 221 |  |
|  | R | GGTACGAATACCGCTGTCA |  |  |  |
| *adeK* | F | TTGATAGTTACTTGACTGTTC |  | 162 |  |
|  | R | GGTTGGTGAACCACTGTATC |  |  |  |

**Table S2: Minimum Inhibitory Concentrations (MICs) values of imipenem and efflux pump inhibitors against 37 CRAb strains.**

| ***A.baumannii* strains** | **Antibiotics and Efflux Pump Inhibitors** | | | | | | | | | | | | | | | |
| --- | --- | --- | --- | --- | --- | --- | --- | --- | --- | --- | --- | --- | --- | --- | --- | --- |
|  | **Imipenem** | | **TMP** | | **OMP** | | **Pantoprazole /esmoprazole** | | **CCCP** | | **Cinnamon oil** | | **Thyme oil** | | **Clove, carraway oils** | |
|  | **aMICs (mg/L)** | **bMBC** | **MIC** | **MBC** | **MIC** | **MBC** | **MIC** | **MBC** | **MIC** | **MBC** | **MIC (%v/v)** | **MBC** | **MIC** | **MBC** | **MIC** | **MBC** |
| **91** | 32 | 32 | 25 | 25 | 25 | 25 | >50 | >50 | >1024 | >1024 | 0.5 | 0.5 | >4 | >4 | >4 | >4 |
| **92** | 16 | 32 | 25 | 25 | 25 | 25 |  |  |  |  | 0.5 | 0.5 | >4 | >4 |  |  |
| **93** | 8 | 8 | 15 | 15 | 25 | 25 |  |  |  |  | 0.25 | 0.25 | >4 | >4 |  |  |
| **94** | 16 | 16 | 15 | 15 | 25 | 25 |  |  |  |  | 0.5 | 0.5 | >4 | >4 |  |  |
| **95** | 16 | 16 | 15 | 15 | 25 | 25 |  |  |  |  | 0.5 | 0.5 | >4 | >4 |  |  |
| **9** | 16 | 16 | 15 | 15 | 25 | 25 |  |  |  |  | 0.5 | 0.5 | >4 | >4 |  |  |
| **97** | 128 | 128 | 15 | 15 | 25 | 25 |  |  |  |  | 2 | 2 | >4 | >4 |  |  |
| **98** | 64 | 64 | 25 | 25 | 20 | 25 |  |  |  |  | 0.5 | 0.5 | >4 | >4 |  |  |
| **99** | 256 | 256 | 25 | 25 | 25 | 25 |  |  |  |  | 0.5 | 0.5 | >4 | >4 |  |  |
| **100** | 1024 | 1024 | 25 | 25 | 25 | 25 |  |  |  |  | 0.5 | 0.5 | >4 | >4 |  |  |
| **70** | 64 | 64 | 15 | 25 | 25 | 25 |  |  |  |  | 0.5 | 0.5 | >4 | >4 |  |  |
| **71** | 64 | 64 | 5 | 5 | 25 | 25 |  |  |  |  | 0.5 | 0.5 | >4 | >4 |  |  |
| **72** | 64 | 64 | 5 | 5 | 25 | 25 |  |  |  |  | 0.5 | 0.5 | 1 | >4 |  |  |
| **73** | 64 | 64 | 15 | 15 | 25 | 25 |  |  |  |  | 1 | 1 | 1 | 1 |  |  |
| **74** | 32 | 32 | 15 | 15 | 25 | 25 |  |  |  |  | 0.5 | 0.5 | 1 | 1 |  |  |
| **75** | 32 | 32 | 25 | 25 | 25 | 25 |  |  |  |  | 0.5 | 0.5 | >4 | >4 |  |  |
| **76** | 32 | 32 | 25 | 25 | 25 | 25 |  |  |  |  | 0.5 | 0.5 | >4 | >4 |  |  |
| **77** | 32 | 32 | 25 | 25 | 25 | 25 |  |  |  |  | 0.5 | 0.5 | >4 | >4 |  |  |
| **78** | 64 | 64 | 25 | 25 | 20 | 20 |  |  |  |  | 0.5 | 0.5 | >4 | >4 |  |  |
| **79** | 64 | 64 | 25 | 25 | 25 | 25 |  |  |  |  | 2 | 2 | >4 | >4 |  |  |
| **80** | 64 | 64 | 25 | 25 | 25 | 25 |  |  |  |  | 0.5 | 0.5 | >4 | >4 |  |  |
| **81** | 32 | 32 | 25 | 25 | 25 | 25 |  |  |  |  | 0.5 | 0.5 | >4 | >4 |  |  |
| **82** | 128 | 128 | 25 | 25 | 25 | 25 |  |  |  |  | 0.5 | 0.5 | >4 | >4 |  |  |
| **83** | 64 | 64 | 25 | 25 | 25 | 25 |  |  |  |  | 0.5 | 0.5 | >4 | >4 |  |  |
| **84** | 128 | 128 | 25 | 25 | 25 | 25 |  |  |  |  | 0.125 | 0.125 | >4 | >4 |  |  |
| **23** | 256 | 256 | 15 | 15 | 25 | 25 |  |  |  |  | 0.5 | 0.5 | >4 | >4 |  |  |
| **16** | 128 | 128 | 15 | 15 | 25 | 25 |  |  |  |  | 0.5 | 0.5 | >4 | >4 |  |  |
| **47** | 64 | 64 | 15 | 15 | 25 | 25 |  |  |  |  | 0.5 | 0.5 | >4 | >4 |  |  |
| **65** | 32 | 32 | 25 | 25 | 25 | 25 |  |  |  |  | 0.5 | 0.5 | >4 | >4 |  |  |
| **54** | 512 | 512 | 5 | 5 | 25 | 25 |  |  |  |  | 0.25 | 0.25 | >4 | >4 |  |  |
| **13** | 512 | 512 | 15 | 15 | 25 | 25 |  |  |  |  | 0.5 | 0.5 | >4 | >4 |  |  |
| **43** | 16 | 32 | 25 | 25 | 25 | 25 |  |  |  |  | 0.25 | 0.25 | >4 | >4 |  |  |
| **17** | 256 | 256 | 15 | 15 | 25 | 25 |  |  |  |  | 0.5 | 0.5 | >4 | >4 |  |  |
| **14** | 128 | 128 | 25 | 25 | 25 | 25 |  |  |  |  | 0.25 | 0.25 | >4 | >4 |  |  |
| **59** | 128 | 128 | 15 | 15 | 25 | 25 |  |  |  |  | 0.5 | 0.5 | >4 | >4 |  |  |
| **1** | 256 | 256 | 15 | 15 | 25 | 25 |  |  |  |  | 0.5 | 0.5 | >4 | >4 |  |  |
| **15** | 16 | 16 | 25 | 25 | 25 | 50 |  |  |  |  | 0.5 | 0.5 | >4 | >4 |  |  |

**^a^MICs: Minimum Inhibitory Concertations.**

**Table** **S3: Chemical composition of cinnamon and thyme essential oils using GC/MS/MS analysis.**

| **No.** | **Cinnamon oil** | | **Area**  **(%)** | **Thyme oil** | | **Area**  **(%)** |
| --- | --- | --- | --- | --- | --- | --- |
|  | **RT** | **Compound** |  | **RT** | **Compound** |  |
| 1 | 4.752 | 4-Terpinenyl acetate. | 1.35 | 9.24 | α-Pinene. | 0.08 |
| 2 | 4.887 | ϒ-Terpinene. | 1.51 | 9.69 | Trans-*p*-Menthane. | 0.04 |
| 3 | 5.347 | β-Myrcene. | 1.17 | 9.74 | β-Pinene. | 1.28 |
| 4 | 5.502 | α-Thujene. | 2.09 | 9.87 | Myrcene. | 0.55 |
| 5 | 5.695 | *p*-cymene. | 2.84 | 10.04 | Pseudolimonene. | 0.02 |
| 6 | 5.785 | **α-Terpinene.** | **6.38** | 10.21 | m-cymene. | 0.15 |
| 7 | 5.962 | **α-Pinene.** | **12.0** | 10.24 | ***p*-cymene**. | **29.05** |
| 8 | 6.409 | 2’-Hydroxy-3,4,5-trimethoxychalcone. | 1.32 | 10.28 | Limonene. | 3.68 |
| 9 | 6.536 | **Linalool.** | **18.54** | 10.31 | Eucalytol. | 0.13 |
| 10 | 7.389 | Terpinen-4-ol. | 3.98 | 10.59 | **ᵞ**- Terpiene. | 5.03 |
| 11 | 8.852 | **Cinnamaldehyde.** | **6.69** | 10.73 | Cis-Linalool oxide. | 0.05 |
| 12 | 8.725 | **Coumarin.** | **33.90** | 10.88 | Trans-linalool oxide (furanoid) | 0.05 |
| 13 | 9.127 | 6-methyl-phenol. | 100 | 11.00 | **Linalool.** | **5.24** |
| 14 | 9.476 | 2-(1,1-dimethylethyl)-5-methyl-Eugenol. | 5.23 | 11.34 | Cis-*p*-mentha-1(7),8-dien-2-ol. | 0.06 |
| 15 | 9.738 | **Longifolene.** | **45.08** | 11.68 | Trans-Verbenol. | 0.17 |
| 16 | 9.853 | Caryophyllene. | 6.23 | 11.78 | Trans- Sabienenehydrate. | 0.91 |
| 17 | 9.103 | **Isoeugenol.** | **15.29** | 12.60 | Trans-*p*-Mentha-2,8-dien-1-ol. | 0.11 |
| 18 | 10.025 | **Cinnamyl alcohol.** | **10.84** | 13.02 | **Thymol.** | **51.80** |
| 19 | 10.091 | α-Guaiene. | 7.11 | 13.07 | Carvacol. | 1.58 |
| 20 | 10.214 | Dimethylfraxetin. | 1.86 |  |  |  |
| 21 | 10.407 | α-Selinene. | 1.66 |  |  |  |
| 22 | 10.517 | 5,7-Dihydroxy-4-methylcoumarin. | 6.60 |  |  |  |
| 23 | 10.71 | Myricetin-3’,5’-dimethy ether. | 2.90 |  |  |  |
| 24 | 10.891 | 3,4,5-Trimethoxycinamic acid. | 2.45 |  |  |  |
| 25 | 11.108 | Cis-Sesquisabinene hydrate. | 3.99 |  |  |  |
| 26 | 11.21 | Ledol. | 1.65 |  |  |  |
| 27 | 11.538 | Nerolidol. | 1.30 |  |  |  |
| 28 | 11.666 | Thunbergol. | 1.22 |  |  |  |
| 29 | 12.309 | Nopol(terpene). | 6.78 |  |  |  |
| 30 | 12.887 | Gardenin. | 1.77 |  |  |  |
| 31 | 13.704 | 3,7,8,2’-Tetramethoxyflavone. | 1.54 |  |  |  |
| 32 | 13.884 | Dihydroquercitrin. | 1.59 |  |  |  |
| 33 | 14.429 | Diethylstilbestrol. | 1.08 |  |  |  |
| 34 | 14.597 | o-Cresol. | 1.76 |  |  |  |
| 35 | 15.75 | Α-phenyl-3,5-di-tert-butyl-4-hydroxyacetophenone. | 3.90 |  |  |  |
| 36 | 16.701 | (-)-Spathulenol. | 1.63 |  |  |  |
| 37 | 17.427 | 3’-Benzyloxy-4,5,6,7-tetramethoxyflavone. | 1.53 |  |  |  |
| 38 | 20.302 | Retinal. | 1.01 |  |  |  |
| 39 | 23.127 | 3-(3,4-Dimethoxyphenyl)-4,7-dimethylcoumarin. | 1.21 |  |  |  |

| **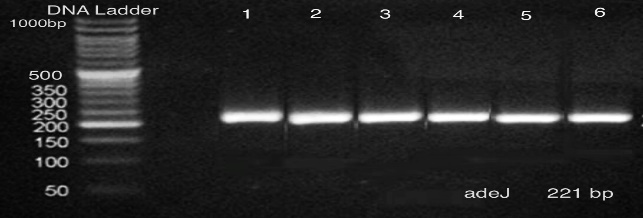a** | **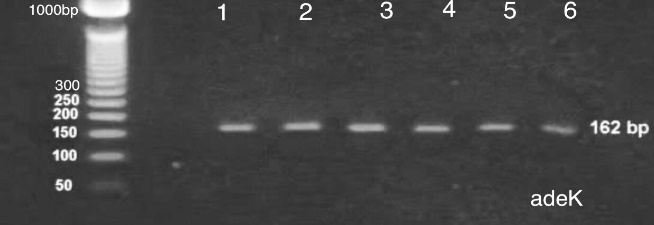b** |
| --- | --- |
| **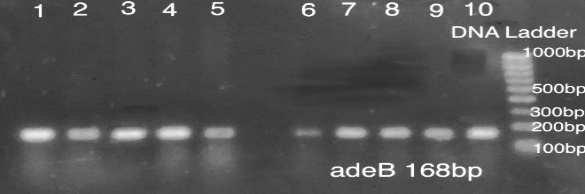c** | **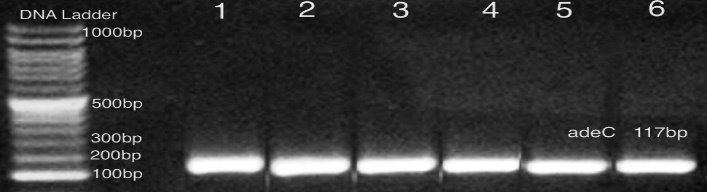d** |

**Figure S1: Electrophoretogram of the PCR amplification of the efflux pump genes in CRAb strains, where a. *adeJ* gene amplified at 221bp; b. *adeK* gene amplified at 162bp; c. *adeB* amplified at 168bp; and d. *adeC* gene amplified at 117bp; DNA ladder; 100-1000bp.**
